# Women’s Hospital

**Published:** 1877-07

**Authors:** 


### Women's Hospital.

OUT IN CIRCULARS,—IF IN BUFFALO, MIGHT BE CALLED "ADVANCED SHEETS."

Drs. Peaslee, Emmett and Thomas publish the particulars of the separation of Dr. J. Marion Sims from the Woman's Hospital, New York. Dr. Sims makes reply to their printed circular, defending his own action and censuring his colleagues for withholding from him support, and the governers for unjustifiable interference in the number of spectators of operations to be admitted upon invitation of surgeons of the hospital, limiting the number to fifteen. He says to the governers as we think truly: " If it is right to admit 15, it is just as right to admit 17, 18 or 20. You might safely have left this to the discrimation and better judgment of the surgeons.

If we do our duty to our patients; if we treat them in a kind and considerate manner; if we give them time and care necessary for their restoration to health; if we give them all advantage of treatment that you yourselves could command in your own homes, we have done our whole duty, and this should end your personal supervision over our actions.

But when you come to invade the sanctity of our operating room, and to dictate to us who shall be present and who shall not be present to witness operations, you evidently over-step the limits of your authority. And when you come to count the number of doctors we invite to witness operations, it seems to me, you are not confining yourselves to your own legitimate sphere." This part of Dr. Sims' speech is truthful and manly, and we admire its tone and spirit. Of the differences between the distinguished members of the staff of the Woman's Hospital, *of* the State of New York, or *in* the State of New York, we have nothing to say. We respect them all too highly to point out any mistakes they may have made. Physicians contribute too largely to hospitals and similar institutions, not to have their feelings respected. The whole hospital system rests in, and depends upon the gratuitious and charitable care of physicians, and the public should long since have learned that hospitals are the institutions of physicians, aided of course by governing boards of benevolent ladies and gentlemen. The medical profession contribute alone more for the care of the sick poor, than all others combined, and it is quite time an appreciating public should recognize and acknowledge this fact, otherwise the great day will dawn, when medical services in hospitals will command adequate pecuniary consideration, instead of doubtful and uncertain honor.

